# Comparative Evaluation of a Nanocellulose Hydrogel and Matrigel^®^ in a Vascularized Intestinal–CAM Model

**DOI:** 10.3390/gels12040278

**Published:** 2026-03-26

**Authors:** Christa Schimpel, Christina Passegger, Kerstin Auer, Herbert Strobl, Waltraud Huber, Nassim Ghaffari-Tabrizi-Wizsy

**Affiliations:** 1 Otto Loewi Research Center for Vascular Biology, Immunology and Inflammation, Division of Immunology, Medical University of Graz, 8010 Graz, Austria; christina.passegger@medunigraz.at (C.P.); herbert.strobl@medunigraz.at (H.S.); 2 Institute of Biomedical Science, University of Applied Sciences, 8020 Graz, Austria; kerstin.auer@edu.fh-joanneum.at; 3 Research Unit CAM Laboratory, Otto Loewi Research Center for Vascular Biology, Immunology and Inflammation, Division of Immunology, Medical University of Graz, 8010 Graz, Austria; waltraud.huber@medunigraz.at (W.H.); nassim.ghaffari@medunigraz.at (N.G.-T.-W.)

**Keywords:** nanocellulose hydrogel, scaffold integrity, matrigel, chorioallantoic membrane (CAM), hydrogel comparison, tissue engineering

## Abstract

Hydrogel-based scaffolds are central to three-dimensional (3D) epithelial culture systems, yet commonly used matrices such as Matrigel^®^ suffer from batch variability, undefined composition, and limited translational relevance. Here, we comparatively evaluated an animal-free nanocellulose hydrogel (GrowDex^®^) and Matrigel^®^ in a hybrid vascularized intestinal–chorioallantoic membrane (CAM) model. Pre-cultured epithelial–immune constructs (Caco-2/HT29-MTX with immune components) were embedded in both matrices and grafted onto the CAM for 72 h. Histological and immunohistochemical analyses revealed that nanocellulose-based constructs maintained more cohesive epithelial coverage, improved scaffold integrity, and yielded a more continuous cytokeratin-positive layer at the scaffold–CAM interface. In contrast, Matrigel^®^ constructs frequently exhibited heterogeneous epithelial distribution and central discontinuities. While both matrices enabled CAM engraftment, the chemically defined nanocellulose hydrogel demonstrated enhanced structural robustness during in vivo exposure and histological processing. These findings highlight the suitability of standardized nanocellulose hydrogels for reproducible scaffold-based epithelial models in vascularized environments.

## 1. Introduction

The human small intestine is a highly specialized organ that combines efficient nutrient absorption with a critical role in immune defense [1,2]. Its mucosal surface is shaped into crypts and villi, maximizing absorptive area and creating a complex architecture composed of diverse epithelial cell types—including enterocytes, goblet cells, and Paneth cells—that interact closely with resident and infiltrating immune cells such as dendritic cells (DCs) and T lymphocytes to maintain barrier integrity and immune homeostasis [3,4].

Traditional 2D cultures have provided mechanistic insights but lack the 3D architecture and cellular diversity of the intestinal mucosa [5]. More advanced 3D epithelial–immune co-cultures better mimic barrier function [6] and immune interactions but remain avascular, limiting viability and physiological relevance [7]. The structural stability and reproducibility of such systems critically depend on the properties of the supporting hydrogel matrix.

The chorioallantoic membrane (CAM) of fertilized chicken eggs provides a naturally vascularized, immune-tolerant environment used in oncology, angiogenesis, and tissue engineering [8,9]. It enables rapid, cost-efficient testing of tissue constructs and biomaterials while aligning with the principles of Replacement, Reduction, and Refinement (3Rs) in animal research [10]. Importantly, the CAM assay enables evaluation of scaffold integrity and host–material interactions under perfused conditions [11]. Beyond its broad use in angiogenesis and tumor biology [12,13,14], the CAM assay has also emerged as a practical platform for the short-term evaluation of biomaterials and tissue constructs under vascularized conditions [15]. In the context of intestinal tissue engineering, CAM-based approaches are particularly valuable because they permit assessment of early graft integration and structural preservation in a perfused environment that cannot be captured in conventional static in vitro culture.

Despite its advantages, successful CAM engraftment of intestinal constructs requires a suitable extracellular matrix (ECM) to maintain epithelial structure, promote host integration, and allow reproducible histological processing. Matrigel^®^, derived from Engelbreth–Holm–Swarm mouse sarcoma, is a commonly used ECM that supports epithelial differentiation. Although widely used, Matrigel^®^ exhibits batch-to-batch variability, undefined growth factor content, and limited translational relevance due to its tumor-derived origin [16,17].

Nanocellulose-based hydrogels have been increasingly used in three-dimensional cell culture and tissue engineering because of their defined composition, fibrillar architecture, and favorable biocompatibility [18]. In addition to their application as bioinks and scaffold materials [19], such hydrogels have been reported to support cell viability, spatial organization, and reproducible culture conditions in a range of experimental systems [20,21,22]. These properties make them attractive candidates for comparative scaffold evaluation in epithelial models.

GrowDex^®^, an animal-free nanocellulose-based hydrogel, provides a standardized and mechanically stable ECM alternative. Formed by entangled cellulose nanofibrils, the hydrogel establishes a physically crosslinked fibrillar network with defined composition and tunable mechanical characteristics [23]. Its animal-free origin and structural stability makes it an attractive candidates for standardized scaffold-based epithelial models [24].

We hypothesized that a chemically defined nanocellulose hydrogel would provide improved structural stability and more consistent epithelial organization under vascularized CAM conditions compared to Matrigel^®^. This hypothesis was based on the defined composition, fibrillar network structure, and reported reproducibility of nanocellulose-based hydrogels, in contrast to the biologically variable and tumor-derived nature of Matrigel^®^. Here, we present a hybrid intestinal–CAM model combining epithelial–immune co-cultures with CAM vascularization. We directly compare GrowDex^®^ and Matrigel^®^ scaffolds, evaluating morphology, epithelial integrity, and handling properties, thereby highlighting its suitability for rapid comparative evaluation of hydrogel performance in vascularized epithelial models.

## 2. Results

### 2.1. Macroscopic Evaluation and CAM Integration

**Figure 1:**
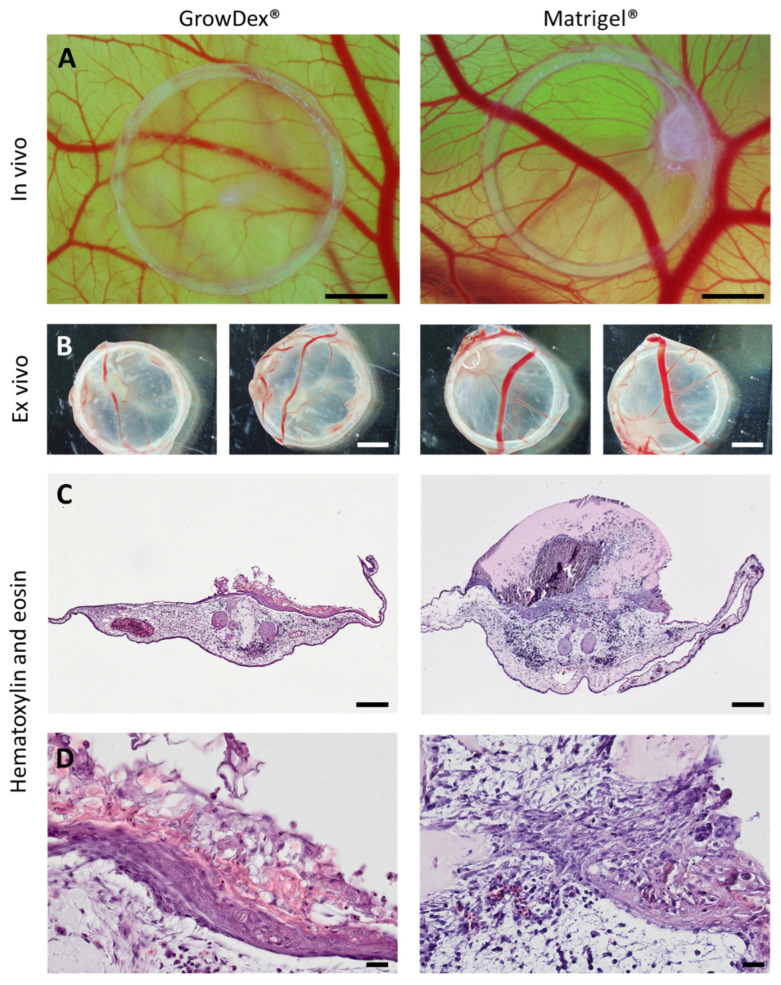
Macroscopic appearance and histological organization of intestinal–CAM constructs embedded in GrowDex^®^ and Matrigel^®^. (**A**) Representative in vivo images of intestinal constructs on the chorioallantoic membrane (CAM) at Day 4 post-transfer, illustrating overall graft morphology and the surrounding vascular network. (**B**) Ex vivo top and bottom views of excised xenografts after 72 h on the CAM, demonstrating graft integrity and vascular imprinting on the construct surface. (**C**) Hematoxylin and eosin (H&E) staining at low magnification showing the overall scaffold architecture and the interface between the epithelial construct and the underlying CAM tissue in GrowDex^®^- and Matrigel^®^-based grafts. (**D**) Higher magnification H&E images highlighting differences in epithelial organization at the scaffold–CAM interface, with more continuous epithelial coverage observed in GrowDex^®^ constructs and more variable coverage with central discontinuities in Matrigel^®^ constructs. Scale bars: (**A**) 2 mm; (**B**) 2 mm; (**C**) 200 µm; (**D**) 20 µm.

After 72 h on the CAM, all grafted constructs remained adherent. Macroscopically, both GrowDex^®^- and Matrigel^®^-based constructs maintained contact with the CAM surface, with no obvious differences in overall vascularization patterns visible at this level. In vivo images at Day 4 document the constructs’ appearance and vascular surroundings, while ex vivo images provide a top- and bottom-up view of the excised xenografts (see Figure 1A,B). In both conditions, the silicone ring-defined circular shape was largely retained, and CAM vessels extended toward the graft periphery. More distinct differences in epithelial coverage and matrix integrity became apparent at the histological level.

### 2.2. Histological Analysis (Hematoxylin & Eosin)

H&E staining revealed material-dependent differences in scaffold integrity and epithelial organization at the graft–CAM interface (see Figure 1C,D).

In GrowDex^®^ constructs, a more extensive and cohesive epithelial layer was observed on the scaffold surface, directly adjacent to the CAM tissue. This layer covered larger areas without major interruptions, and the interface with the underlying CAM—containing recognizable blood vessels—appeared compact, without discernible gaps. The overall scaffold integrity remained preserved in GrowDex^®^ constructs under CAM conditions.

In Matrigel^®^ constructs, the cellular coverage at the scaffold surface was more variable. While peripheral regions sometimes showed denser coverage, central areas often exhibited reduced cell density or discontinuous layers. The scaffold material in these areas appeared less compact, and separation artifacts between graft and CAM tissue occurred more frequently. Although CAM vasculature was visible in both scaffold types, the continuity and cohesion of the epithelial layer at the host–graft interface were overall better preserved in GrowDex^®^.

Due to matrix-specific differences in structure and thickness, exact visual appearance may vary between sections; however, image acquisition and evaluation were performed under comparable conditions.

### 2.3. Immunohistochemistry (Cytokeratin AE1/AE3)

**Figure 2:**
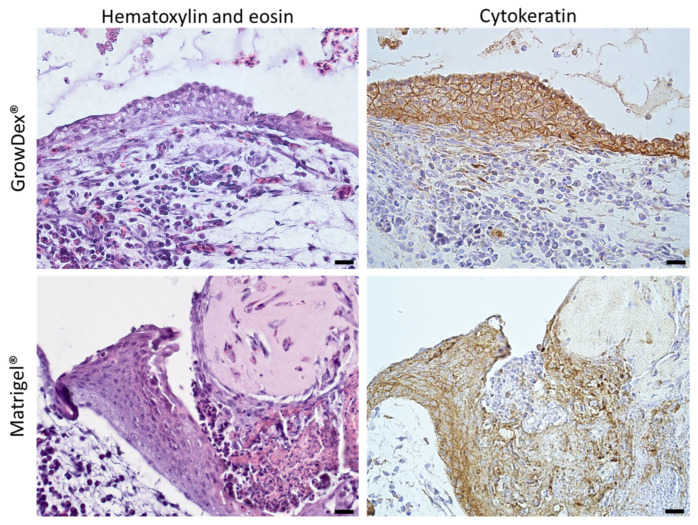
Epithelial organization in the hybrid intestinal–CAM model. Representative paraffin sections of CAM-grafted intestinal constructs stained for H&E and cytokeratin AE1/AE3. (**Upper panels**) show GrowDex^®^-based constructs, displaying a continuous, surface-aligned epithelial layer with uniform cytokeratin expression along the scaffold–CAM interface. (**Lower panels**) show Matrigel^®^-based constructs, in which cytokeratin-positive epithelial cells are distributed more heterogeneously, with disrupted coverage and gaps, particularly in central regions. Images illustrate both overall tissue architecture and higher magnification views highlighting differences in epithelial continuity between the two scaffold types. Scale bars: 20 µm.

Cytokeratin AE1/AE3 staining (Figure 2) confirmed the histologically observed material-dependent differences in epithelial organization. In GrowDex^®^ constructs, staining was strong and continuous along the surface-facing layer across large areas of the graft. At higher magnifications, the epithelial signal appeared evenly distributed, forming a well-defined boundary between the scaffold and the CAM tissue. This pattern indicates that GrowDex^®^ supported the development of cohesive, polarized epithelial coverage.

In Matrigel^®^ constructs, cytokeratin-positive cells were also detected but showed a more heterogeneous distribution. Peripheral regions tended to display stronger and more consistent staining, while central areas frequently exhibited weaker or interrupted signal. In some sections, staining appeared patchy, with cytokeratin expression limited to isolated cell clusters rather than forming a continuous layer.

Overall, epithelial organization and marker expression were more consistent in GrowDex^®^, aligning with the structural observations from H&E staining.

### 2.4. Matrix Handling and Processing Compatibility

Both matrices required careful handling during transfer due to their soft, gel-like consistency. No pronounced differences in handling stability during transfer were observed. In some instances, Matrigel^®^ appeared slightly more stable when lifted with the silicone ring; however, GrowDex^®^ constructs exhibited improved structural preservation during subsequent histological processing.

### 2.5. Semi-Quantitative Assessment of Epithelial Continuity

To support the descriptive histological observations, a semi-quantitative image analysis was performed on low-magnification H&E images (4× objective) containing the entire scaffold–CAM interface. Epithelial coverage was calculated as the fraction of the scaffold interface length covered by epithelial cells relative to the total interface length. GrowDex^®^ constructs showed significantly higher epithelial coverage compared with Matrigel^®^ constructs (48.1 ± 13.8% vs. 21.3 ± 2.5%, Mann–Whitney test, *p* = 0.029). These results were consistent with the qualitative observations from H&E and cytokeratin staining, indicating a more homogeneous epithelial organization in the nanocellulose-based scaffold.

## 3. Discussion

This study establishes a hybrid intestinal–CAM model that enables the evaluation of epithelial constructs within a naturally vascularized host environment. Within this setting, both GrowDex^®^ and Matrigel^®^ supported graft adhesion and short-term survival over the 72 h incubation period. However, histological and immunohistochemical analyses revealed clear differences in epithelial organization and scaffold integrity between the two matrices.

In GrowDex^®^ constructs, the epithelial coverage at the scaffold–CAM interface was more consistent, with large areas showing uninterrupted, dense cell layers in close apposition to the host tissue. Cytokeratin AE1/AE3 staining further confirmed a more uniform epithelial distribution along the surface-facing layer. These findings align with the nanocellulose-based structure of GrowDex^®^, which provides a mechanically stable, chemically defined, and animal-free hydrogel network that supports epithelial adhesion, nutrient diffusion and spatial organization under CAM conditions. The preserved epithelial architecture, including in central scaffold regions, suggests improved structural stability of the construct following grafting. In addition, nanocellulose-based hydrogels are generally regarded as biocompatible materials and have been widely explored in experimental tissue engineering applications [21]. Although the present study did not specifically investigate nanofibril safety endpoints, the observed epithelial preservation and CAM compatibility support their suitability for short-term scaffold-based modelling in this setting.

In contrast, Matrigel^®^ constructs frequently displayed heterogeneous epithelial distribution, with reduced cell density or discontinuous coverage in central regions. Cytokeratin staining appeared patchy and was often restricted to peripheral areas of the graft. Such variability is consistent with the undefined composition and batch-dependent characteristics of tumor-derived ECM substitutes, which may influence matrix integrity and epithelial organization during CAM engraftment.

The CAM assay proved to be a rapid and technically accessible platform for short-term assessment of scaffold performance in a perfused biological environment, consistent with its established role in biomaterial evaluation [25]. While limited to early integration events, this model enables direct comparison of hydrogel stability, host–material interaction, and epithelial continuity within a defined and reproducible timeframe.

The co-culture system included CD34^+^ progenitor cells and CD4^+^ T cells to preserve the established hybrid intestinal model and to better reflect the multicellular composition of the intestinal microenvironment [6]. However, the functional contribution of these immune cell populations was not the focus of the present scaffold comparison and was therefore not analyzed separately. Future studies may address whether matrix-dependent effects also influence immune cell localization or epithelial–immune crosstalk.

The present study has several limitations. First, the CAM assay is inherently restricted to a short-term observation window and therefore captures early integration events rather than long-term tissue maturation. Second, the present work was designed as a comparative proof-of-concept study and therefore relies primarily on histological and immunohistochemical endpoints, complemented by semi-quantitative image analysis. Third, physicochemical hydrogel parameters such as storage modulus, pore structure, and diffusion behavior were not measured directly in this study.

From a translational perspective, chemically defined and animal-free hydrogels offer advantages regarding standardization, regulatory compatibility, and experimental reproducibility. The present findings suggest that nanocellulose-based matrices may represent promising and structurally stable alternatives to Matrigel^®^ for scaffold-based epithelial models in vascularized settings.

## 4. Conclusions

This study shows that, within the limits of a short-term CAM-based comparison, a nanocellulose-based hydrogel was associated with improved scaffold preservation and more consistent epithelial continuity than Matrigel^®^ in a vascularized intestinal–CAM model. The presented hybrid platform provides a practical approach for the comparative evaluation of hydrogel performance under perfused biological conditions. Chemically defined, animal-free hydrogels such as GrowDex^®^ may therefore represent promising alternatives to tumor-derived ECM substitutes for scaffold-based epithelial models.

## 5. Materials and Methods

### 5.1. Cell Lines and Co-Culture Preparation

Epithelial cells: Caco-2 (ACC169, HTB-37 clone; German Collection of Microorganisms and Cell Cultures) and HT29-MTX cells (kindly provided by T. Lesuffleur (INSERM UMR S 938, Paris, France) were thawed in a 37 °C water bath until the pellet was fully melted. To minimize osmotic stress, ~1 mL pre-warmed medium was added dropwise before transfer to a 50 mL tube, followed by centrifugation (1250 rpm, 5 min). Pellets were resuspended in complete DMEM (Thermo Fisher Scientific, Vienna, Austria) supplemented with 10% FBS, 1% NEAA, 1% penicillin–streptomycin and maintained at 37 °C, 5% CO_2_. Passaging was performed at 70–80% confluence using trypsin–EDTA, neutralization with medium, centrifugation, and reseeding. All experiments used Caco-2/HT29-MTX co-cultures only.

CD34^+^ hematopoietic progenitor cells: Umbilical cord blood samples were collected from healthy full-term deliveries after written informed consent and approval by the Ethics Committee of the Medical University of Graz (approval date: 18 July 2014). Mononuclear cells were isolated by Ficoll density gradient centrifugation, and CD34^+^ hematopoietic progenitor cells were purified using magnetic separation (EasySep™ Human Cord Blood CD34 Positive Selection Kit II, STEMCELL Technologies, Grenoble, France) according to the manufacturer’s instructions. Prior to use in co-culture experiments, cells were resuspended in X-VIVO^®^ 15 medium (Lonza, Basel, Switzerland) with GlutaMAX, penicillin–streptomycin (Thermo Fisher Scientific, Vienna, Austria), and SCF/FLT3-L/TPO (PeproTech, Rocky Hill, NJ, USA; 50 ng/mL each), centrifuged (1280 rpm, 5 min), and cultured for 5 days at 37 °C/5% CO_2_ before co-culture use.

CD4^+^ T cells: T cells were isolated from peripheral blood buffy coats using a negative selection magnetic enrichment kit (MagniSort™ Human CD4 T Cell Enrichment Kit, Thermo Fisher Scientific, Vienna, Austria) according to the manufacturer’s protocol. Cells were washed twice in serum-free RPMI, resuspended in pre-warmed complete DMEM, counted, and directly used for co-culture setup.

### 5.2. Scaffold Preparation of Epithelial–Immune Co-Culture Constructs

Caco-2 and HT29-MTX cells (7:3 ratio) were combined with 3 × 10^5^ CD4^+^ T cells and 3 × 10^5^ CD34^+^ progenitors per well, then embedded in GrowDex^®^ (UPM Biomedicals, Helsinki, Finland; 0.5% *w*/*v*) or Matrigel^®^ (Corning, Corning, NY, USA; on ice, manufacturer’s protocol). Cell–matrix mixtures were prepared immediately before use. Constructs were seeded into silicone rings (6 mm inner diameter) in 24-well plates and pre-cultured for 7 days under standard conditions to allow epithelial organization and immune cell incorporation before CAM transfer.

### 5.3. CAM Assay Setup

Fertilized Lohmann White chicken eggs were incubated horizontally at 37.6 °C and 60% relative humidity. On embryonic day 3 (ED3), eggs were cracked, and embryos were transferred ex ovo into sterile weigh boats. On ED10, epithelial constructs were carefully placed onto the CAM surface using fine forceps and stabilized with silicone rings. Embryos were incubated for an additional 72 h post-grafting. The 72 h post-grafting observation window was selected to assess early scaffold integration and epithelial preservation within the commonly used experimental timeframe of the CAM assay.

### 5.4. Fixation, Embedding, and Sectioning

On ED13, xenografts were excised, fixed in 4% paraformaldehyde (24 h, RT), dehydrated in ethanol, cleared in xylene, and paraffin-embedded. Sections (5 µm) were cut using a rotary microtome.

### 5.5. Histology and Immunohistochemistry

H&E staining: Sections were deparaffinized, rehydrated, stained in Mayer’s hematoxylin (4 min), washed, counterstained with eosin (2 min), dehydrated, cleared, and mounted.

Immunohistochemistry: Cytokeratin AE1/AE3 (mouse anti-human, Dako, Agilent Technologies, Santa Clara, CA, USA; 1:100 in TBST) was used. Sections underwent deparaffinization, heat-mediated antigen retrieval, peroxidase blocking, protein blocking, primary antibody incubation (1 h, RT), HRP polymer detection, chromogen development, hematoxylin counterstaining, and mounting. All steps followed standard protocols optimized for paraffin sections.

### 5.6. Microscopy

Stained sections were examined with an Olympus BX51 microscope (Olympus, Tokyo, Japan) at 4×, 40× and 400× magnification. Parameters included epithelial continuity, cytokeratin expression, and matrix–CAM interface integrity. Analyses were based on sections obtained from multiple independent biological replicates per condition. Representative images were selected from sections showing the scaffold–CAM interface and were acquired under comparable microscope settings and focal conditions.

### 5.7. Semi-Quantitative Image Analysis

To complement the qualitative histological evaluation, semi-quantitative image analysis was performed on H&E-stained sections using Fiji/ImageJ software (version 1.54p, NIH, USA). Low-magnification images (4× objective) containing the entire scaffold–CAM interface were used for analysis. For each condition, sections from four independent constructs (*n* = 4 per group) were evaluated. Epithelial coverage was calculated as the fraction of the scaffold interface length covered by epithelial cells relative to the total scaffold–CAM interface length. Regions with sectioning artifacts or disrupted tissue were excluded from analysis. Measurements were performed under identical image acquisition settings and averaged to obtain one value per construct. Data are presented as mean ± SD, and statistical comparisons between groups were performed using a Mann–Whitney U test, with *p* < 0.05 considered statistically significant.

### 5.8. Ethics

Umbilical cord blood was obtained from healthy donors during full-term delivery at the University Clinic for Gynaecology and Obstetrics (Frauenklinik), Hospital Graz, Austria. Ethical approval: 26-520 ex 13/14.

Buffy coats were purchased from the Department of Transfusion Medicine, Hospital Graz, Austria.

## Data Availability

All data supporting the findings of this study are included within the article.
